# The prognostic value of systemic inflammatory indices in elderly patients with spontaneous intracerebral hemorrhage

**DOI:** 10.1371/journal.pone.0348944

**Published:** 2026-05-15

**Authors:** Ömer Jaradat, Burak Şahin, Hacı Mehmet Çalışkan

**Affiliations:** 1 Necmettin Erbakan University, Meram Faculty of Medicine, Department of Emergency Medicine, Konya, Turkey; 2 Kırşehir Training and Research Hospital, Clinic of Emergency Medicine, Kırşehir, Turkey; 3 Kırşehir Ahi Evran University, Faculty of Medicine, Department of Emergency Medicine, Kırşehir, Turkey; Universitatsklinikum Regensburg, GERMANY

## Abstract

**Background:**

Very old patients represent the fastest-growing age subgroup among patients with intracerebral hemorrhage (ICH), which justifies studying age-specific prognostic behavior in this population. While systemic inflammatory indices are increasingly recognized for their prognostic potential in various conditions, their specific value in this rapidly expanding geriatric cohort remains unclear. This study aimed to evaluate whether specific indices, particularly the Monocyte-to-Albumin Ratio (MAR) and Systemic Inflammatory Response Index (SIRI), provide independent prognostic information for case-fatality in this population.

**Methods and findings:**

We conducted a retrospective observational cohort study of patients aged ≥65 years with spontaneous ICH admitted to a single tertiary emergency department between 2020 and 2025. Of 326 patients initially screened, 118 consecutive patients met inclusion criteria and constituted the cohort. Inflammatory indices were calculated from admission blood tests. Admission non-contrast cranial computed tomography scans were utilized to measure initial hematoma volume using the validated ABC/2 and ABC/2-derived compartmental methods. The primary outcome was 30-day all-cause case-fatality; secondary outcomes included 48-hour and 90-day case-fatality. Multivariable logistic and Cox regression models were adjusted for age, sex, hemorrhage type, and treatment type. Receiver operating characteristic (ROC) analysis was performed to assess predictive performance.

Both MAR and SIRI were significantly higher in the deceased patient group at 30 days (p = 0.049 and p = 0.029, respectively). ROC analysis showed modest predictive value for 30-day case-fatality (MAR AUC = 0.606; SIRI AUC = 0.617). In multivariable analysis, MAR > 0.018 was independently associated with increased odds of 30-day (OR=3.57, 95% CI: 1.44–8.85) and 90-day case-fatality (OR=3.18, 95% CI: 1.28–7.92), while SIRI was not significant. Cox regression confirmed that patients with MAR ≤ 0.018 had a significantly lower 90-day case-fatality risk (HR = 0.44, 95% CI: 0.26–0.74). Multiple linear regression analysis identified MAR as the sole significant independent predictor of initial hematoma volume (p = 0.037), whereas other indices showed no such association. Furthermore, logistic regression confirmed that each one-unit (1 mL) increase in hemorrhage volume significantly elevated the odds of case-fatality by 9.8% (OR=1.098,95%CI:1.058–1.140,p < 0.001). Study limitations include its retrospective single-center design, and sample size constraints that limited multivariable modeling.

**Conclusions:**

In elderly patients with spontaneous ICH, admission MAR is independently associated with short- and medium-term case-fatality, whereas SIRI offers limited additional prognostic value. MAR may serve as a useful adjunctive marker for risk stratification, but its data-driven cut-offs and modest discrimination necessitate external validation and prospective assessment before clinical adoption.

## Introduction

Spontaneous intracerebral hemorrhage (ICH) accounts for approximately 10–15% of all stroke cases and is characterized by bleeding within the brain parenchyma in the absence of trauma [1]. The global incidence of spontaneous ICH is estimated at 24.6 per 100,000 individuals, with the risk increasing significantly among those aged 55 and older [1,2]. The etiology of ICH is fundamentally multifactorial, involving a complex interplay between predisposing factors, underlying pathologies, and precipitating factors. Most cases are attributable to cerebral small vessel diseases, specifically arteriolosclerosis and cerebral amyloid angiopathy, while macrovascular abnormalities, such as aneurysms and arteriovenous malformations, account for approximately 15% of hemorrhages [1,3]. Within this framework, hypertension is recognized as the strongest modifiable predisposing factor, as it drives the structural progression of arteriolosclerosis. Conversely, elements like anticoagulant therapy are classified as precipitating factors that may trigger clinical hemorrhage in patients with pre-existing underlying pathology [3]. While addressing modifiable predisposing factors, such as hypertension, smoking, and alcohol consumption, is the cornerstone of primary prevention, the clinical prognosis remains poor once a hemorrhage occurs, with 30-day case-fatality rates reaching up to 40% in contemporary series [4,5]. This underscores the need for improved acute-phase prognostication and management, particularly in high-risk populations such as the elderly. Prognosis is closely associated with factors such as hematoma volume, localization (particularly involving the brainstem or basal ganglia), and the patient’s clinical status at presentation [4]. While acute-phase interventions, including blood pressure control and surgical hematoma evacuation, may reduce case-fatality, the incidence of long-term neurological sequelae remains high [4]. Recent research has highlighted the pivotal role of neuroinflammation in secondary brain injury following ICH [6].

After hemorrhage, peripheral immune cells such as neutrophils and monocytes interact with resident microglia, leading to secondary injury through inflammation and disruption of the blood-brain barrier (BBB) [7–9]. Consequently, systemic inflammatory markers, particularly neutrophil-to-lymphocyte ratio (NLR), monocyte-to-lymphocyte ratio (MLR), platelet-to-lymphocyte ratio (PLR), C-reactive protein (CRP)-to-albumin ratio (CAR), monocyte-to-albumin ratio (MAR), systemic immune-inflammation index (SII), systemic inflammatory response index (SIRI), and aggregate index of systemic inflammation (AISI) have emerged as potential indirect indicators of neuroinflammation severity [8–11].

The ease of measuring systemic inflammatory indices in clinical settings makes them promising candidates for predicting case-fatality and functional outcomes in patients with ICH [9,11]. The relevance of these markers is particularly significant in “very old” populations, where age-specific physiological responses and comorbidities necessitate more refined prognostic tools. Recent multicenter data in patients aged ≥80 years have demonstrated that SIRI, when integrated into clinical nomograms, serves as a robust independent predictor of short-term functional outcomes [12]. Similarly, in a cohort of patients aged ≥75 years with lobar ICH, which is a subtype increasingly prevalent in the oldest-old, higher admission NLR and blood glucose levels have emerged as independent predictors of 30-day case-fatality [13]. Furthermore, while traditional clinical determinants such as the Glasgow Coma Scale (GCS) score and hematoma volume remain cornerstone predictors for patients aged ≥65 years, the addition of systemic immune markers provides a more comprehensive assessment of the inflammatory-nutritional response in geriatric populations [14]. In the geriatric population, age-related changes in the immune response and the presence of multiple comorbidities may affect the prognostic value of these indices [1]. Despite growing clinical evidence supporting their prognostic potential, age-specific cut-off values for these inflammatory markers remain undefined in the literature [7]. This study aims to evaluate the prognostic significance of inflammatory indices, including NLR, MLR, and SII, in elderly patients with spontaneous ICH, thereby addressing a critical gap in the existing literature.

The primary outcome of this study was 30-day all-cause case-fatality, while 90-day case-fatality was assessed as a secondary outcome. We considered MAR and SIRI as the indices of primary interest based on biologic plausibility and previous reports; NLR, MLR, PLR, SII, AISI, and CAR were considered exploratory measures.

## Materials and methods

### Study setting and design

This retrospective observational cohort study was conducted in the Emergency Department (ED) of Kırşehir Ahi Evran University Training and Research Hospital. The study commenced following approval by Kırşehir Ahi Evran University Medical Faculty Ethics Committee (Approval No: 2025-09/93, date: 13.05.2025). Reporting adheres to the STROBE statement for observational studies, with items pertinent to model performance according to TRIPOD where applicable. Patients diagnosed with ICH in the ED between April 1, 2020, and April 1, 2025, were included.

### Measurement of inflammatory indices

All indices are calculated using biochemical and complete blood count parameters as follows: NLR: neutrophil count (×10³/μL)/lymphocyte count (×10³/μL); PLR: platelet count (×10³/μL)/lymphocyte count (×10³/μL); MLR: monocyte count (×10³/μL)/lymphocyte count (×10³/μL); MAR: monocyte count (×10³/μL)/albumin (g/dL); CAR: CRP (mg/L)/serum albumin (g/dL); SII: platelet count × neutrophil count/lymphocyte count; SIRI: neutrophil count × monocyte count/ lymphocyte count; AISI: neutrophil count × platelet count × monocyte count/ lymphocyte count.

### Radiological evaluation and volumetric analysis

Admission non‑contrast cranial computed tomography (CT) scans were used for all volume measurements. Slice thickness was 5 mm for all scans. All measurements were performed by a single radiologist blinded to clinical outcomes.

For intraparenchymal hemorrhage (IPH) and the parenchymal component of mixed subarachnoid hemorrhage (SAH) and IPH, volume was calculated using the validated ABC/2 ellipsoid method [15]. The slice with the largest hemorrhage area was identified. The largest diameter (A) and its perpendicular diameter (B) were measured on that slice. The vertical height (C) was obtained by multiplying the number of slices containing hemorrhage by slice thickness (5 mm). For slices with hemorrhage area between 25% and 75% of the largest area, half a slice was counted. Volume (mL) was then A × B × C/ 2.

For isolated SAH and the SAH component of mixed SAH and IPH, we used the ABC/2‑derived compartmental method validated by Föttinger et al. (2024) [16]. SAH blood was measured in five predefined cisternal compartments: prepontine cistern, perimesencephalic cistern, suprasellar cistern, left and right Sylvian fissures (measured separately), and anterior interhemispheric fissure. For each compartment, the maximal thickness (A), maximal length (B), and vertical height (C = number of slices with SAH × slice thickness) were measured. The volume of each compartment was calculated as A × B × C/ 2, and the five compartment volumes were summed to obtain total SAH volume. Intraventricular hemorrhage was not included in this SAH volume measurement, as per the original method [16].

### Study population

Potential participants were screened based on predefined eligibility criteria. The inclusion criteria were: (1) age 65 years or older; (2) diagnosis of non-traumatic ICH via CT; (3) presentation to the ED within 24 hours of symptom onset; and (4) confirmation of diagnosis by both emergency medicine specialists and neurologists. To ensure that the MAR and other inflammatory indices specifically reflected the stroke-related inflammatory state, we applied rigorous exclusion criteria: (1) ICH secondary to trauma; (2) known bleeding diathesis or hematologic disorders; (3) hepatic insufficiency; and (4) age under 65 years. Additionally, patients with incomplete medical records or those who relocated out of the region during the follow-up period were excluded to ensure the feasibility of longitudinal data collection and to minimize attrition bias.

### Data collection

Patient data were retrospectively collected from the hospital’s electronic medical record system between June 1, 2025, and September 1, 2025. All patient identifiers were removed before analysis, and authors had no access to identifiable information during data analysis. Biochemical and hematological parameters of patients diagnosed with ICH were recorded after a thorough review of their medical files. All blood samples were obtained within the first 2 hours of ED presentation, with a maximum allowable interval of 24 hours from symptom onset to arrival, representing the earliest available time point after symptom onset. Furthermore, data regarding all medications used and comorbidities were retrieved from the electronic health records. As our hospital is the sole healthcare provider in the region, access to the patients’ prior and subsequent medical records was feasible. Patients whose medical records were incomplete or who relocated out of the region during the follow-up period were excluded from the study.

### Outcomes

The prespecified primary outcome was 30‑day all‑cause case-fatality. Secondary outcomes included 48‑hour and 90‑day all‑cause case-fatality. Case-fatality status was ascertained from hospital records and regional health information systems.

### Statistical Analysis

Statistical analyses were performed using IBM SPSS Statistics 21.0 software (IBM Corp., Armonk, NY, USA). The normality of data distribution was assessed using the Shapiro-Wilk test. Data are presented as counts (percentages). Continuous variables that showed significantly non-normal (right-skewed) distributions (p < 0.05) are presented as median (interquartile range, IQR), and non-parametric tests were used for between-group comparisons. Parametric tests were used only for variables that were normally distributed, and those results are presented as mean ± standard deviation (SD). The following statistical tests were utilized: the Kruskal-Wallis test, the Mann-Whitney U test, the Chi-square test, Receiver Operating Characteristic (ROC) curve analysis, binary logistic regression analysis, and Cox regression analysis. The Youden Index was used to determine optimal cut-off values. A p-value of < 0.05 was considered statistically significant.

## Results

**Fig 1:**
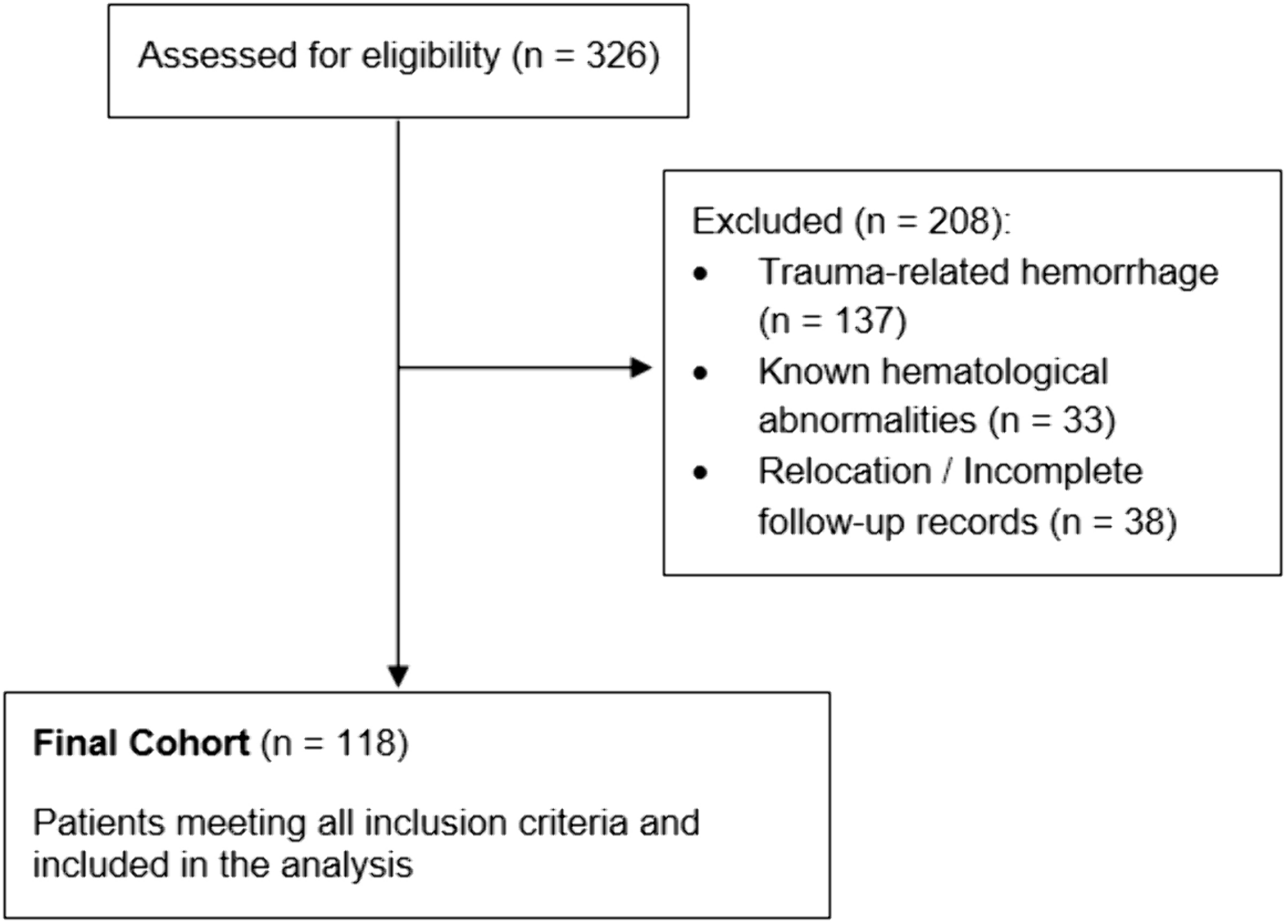
Participant flowchart.

Based on our hospital’s electronic medical records, a total of 326 geriatric patients initially met the diagnosis of ICH during the study period. However, 137 patients were excluded due to trauma-related hemorrhage, 33 were excluded due to known hematological abnormalities, and 38 were excluded because they had moved out of the region during follow-up, making longitudinal data collection unfeasible. Ultimately, 118 patients with complete data and follow-up records were included in the final analysis. The detailed screening process and the specific number of patients excluded at each stage are illustrated in the Participant Flowchart (Fig 1).

A total of 118 patients with spontaneous ICH were included in the study, with a mean age of 74.38 ± 7.71 years (range: 65–95 years). IPH constituted the majority of the cohort (n = 102, 86.4%), followed by isolated SAH (n = 9, 7.6%) and mixed SAH + IPH (n = 7, 5.9%). Regarding acute management, 9 patients (7.6%) underwent surgical intervention, while 109 (92.4%) were managed conservatively. When analyzing baseline characteristics by hemorrhage type, no statistically significant differences were observed regarding gender, age, or inflammatory indices. However, hypertension prevalence was significantly higher in the mixed SAH + IPH group (p = 0.033). Overall, there were 98 survivors and 20 deceased patients (16.9%) at 48 hours, 66 survivors and 52 deceased patients (44.1%) at 30 days, and 62 survivors and 56 deceased patients (47.5%) at the 90-day follow-up. Notably, 30-day case-fatality was significantly higher in the SAH + IPH group (p = 0.032), while 48-hour and 90-day case-fatality rates did not differ significantly between the three hemorrhage subtypes (Table 1).

**Table 1:** Sociodemographic characteristics and parameters by hemorrhage type.

Variables	Hemorrhage type n(%) veya Mean±S.D.			p-value
	SAH (n = 9)		IPH (n = 102)	SAH + IPH (n = 7)
**Gender (F/M), n**	5/4	40/62	4/3	0.458
**Age, years (Median IQR)**	74(16)	73(12)	72(16)	0.635
**NLR**	2.30 (7.02)	3.88 (7.26)	4.90 (2.42)	0.602
**MLR**	0.26 (0.34)	0.37 (0.32)	0.42 (0.29)	0.435
**PLR**	96.53 (101.08)	140.16 (132.86)	103.50 (99.56)	0.119
**MAR**	0.015 (0.01)	0.014 (0.01)	0.021 (0.01)	0.165
**CAR**	0.055 (0.14)	0.10 (0.25)	0.08 (0.92)	0.540
**SII**	479.78 (1387.90)	830.23 (1872.35)	1215.15 (703.57)	0.341
**SIRI**	1.31 (5.16)	2.36 (4.26)	2.84 (3.21)	0.311
**AISI**	230.68 (1023.80)	505.12 (1120.37)	883.72 (815.43)	0.202
**Treatment type, n (%)**				
Surgical	0 (0.0%)	8 (7.8%)	1 (14.3%)	0.649
Conservative	9 (100.0%)	94 (92.2%)	6 (85.7%)	
**48-hour case-fatality, n (%)**	3 (33.3)	15 (14.7)	2 (28.6)	0.247
**30-day case-fatality, n (%)**	3 (33.3)	43 (42.2)	6 (85.7)*	**0.032**
**90-day case-fatality, n (%)**	4 (44.4)	46 (45.1)	6 (85.7)	0.116

SAH: Subarachnoid hemorrhage, IPH: Intraparenchymal hemorrhage, F: Female, M: Male, NLR: Neutrofil/Lenfosit ratio, MLR: Monosit/Lenfosit ratio, PLR: Platelet/Lenfosit ratio, MAR: Monosit/Albumin ratio, CAR: C-reactive protein/Albumin Ratio, SII: Systemic immune-inflammatory index, SIRI: Systemic inflammatory response index, AISI: Aggregate index of systemic inflammation. Continuous variables presented as Mean ± SD (age) or Median (IQR) (inflammatory indices). Categorical variables as n (%). * indicates statistical significance.

When inflammatory indices were analyzed according to case-fatality time points, no significant differences were observed for the 48-hour outcome (Table 2). However, for the 30-day outcome (Table 2), deceased patients had significantly higher NLR (p = 0.037), MAR (p = 0.049), and SIRI (p = 0.029) values than survivors. For the 90-day outcome (Table 3), none of the indices reached statistical significance. These findings suggest a potential association between elevated inflammatory indices and intermediate-term case-fatality.

**Table 2:** Comparison of admission systemic inflammatory indices according to short-term case-fatality outcomes (48 hours and 30 days).

Variables	48-hour case-fatality Median (IQR)		
	Survivors Median (IQR)	Deceased Median (IQR)	p-value
NLR	3.38 (5.75)	7.54 (9.20)	0.159
MLR	0.33 (0.30)	0.41 (0.40)	0.300
PLR	130.72 (124.18)	145.35 (133.48)	0.701
MAR	0.014 (0.01)	0.018 (0.01)	0.287
CAR	0.102 (0.22)	0.108 (0.32)	0.937
SII	795.35 (1729.15)	1214.66 (2584.49)	0.293
SIRI	2.21 (3.89)	3.46 (6.61)	0.208
AISI	498.85 (1028.44)	768.34 (1642.05)	0.432
	**30-day case-fatality**		
	**Survivors** **Median (IQR)**	**Deceased** **Median (IQR)**	**p-value**
NLR	2.90 (5.21)	5.04 (8.83)	0.037*
MLR	0.30 (0.30)	0.41 (0.33)	0.197
PLR	139.82 (132.61)	134.25 (128.88)	0.558
MAR	0.014 (0.01)	0.017 (0.01)	0.049*
CAR	0.11 (0.25)	0.07 (0.21)	0.292
SII	634.28 (1846.4)	1174.74 (1417.93)	0.221
SIRI	1.67 (3.26)	3.22 (5.33)	0.029*
AISI	414.95 (1052.75)	707.05 (1226.42)	0.137

NLR: Neutrofil/Lenfosit ratio, MLR: Monosit/Lenfosit ratio, PLR: Platelet/Lenfosit ratio, MAR: Monosit/Albumin ratio, CAR: C-reactive protein/Albumin Ratio, SII: Systemic immune-inflammatory index, SIRI: Systemic inflammatory response index, AISI: Aggregate index of systemic inflammation. Continuous variables presented as Mean ± SD (age) or Median (IQR) (inflammatory indices). Categorical variables as n (%). * indicates statistical significance. All values are presented as median (interquartile range). Significant p-values (<0.05) are shown in bold.

**Table 3:** Comparison of admission systemic inflammatory indices according to the longer-term case-fatality outcome (90 days).

	90-day case-fatality		
	Survivors Median (IQR)	Deceased Median (IQR)	p-value
NLR	3.05 (5.47)	4.90 (8.71)	0.178
MLR	0.32 (0.32)	0.39 (0.30)	0.394
PLR	144.57 (147.13)	126.24 (122.59)	0.234
MAR	0.014 (0.01)	0.016 (0.01)	0.175
CAR	0.11 (0.26)	0.08 (0.21)	0.449
SII	658.60 (1839.68)	1099.16 (1456.20)	0.710
SIRI	1.76 (3.95)	2.79 (5.13)	0.123
AISI	460.34 (1048.81)	656.97 (1086.25)	0.586

NLR: Neutrofil/Lenfosit ratio, MLR: Monosit/Lenfosit ratio, PLR: Platelet/Lenfosit ratio, MAR: Monosit/Albumin ratio, CAR: C-reactive protein/Albumin Ratio, SII: Systemic immune-inflammatory index, SIRI: Systemic inflammatory response index, AISI: Aggregate index of systemic inflammation. Continuous variables presented as Mean ± SD (age) or Median (IQR) (inflammatory indices). Categorical variables as n (%). All values are presented as median (interquartile range).

**Fig 2:**
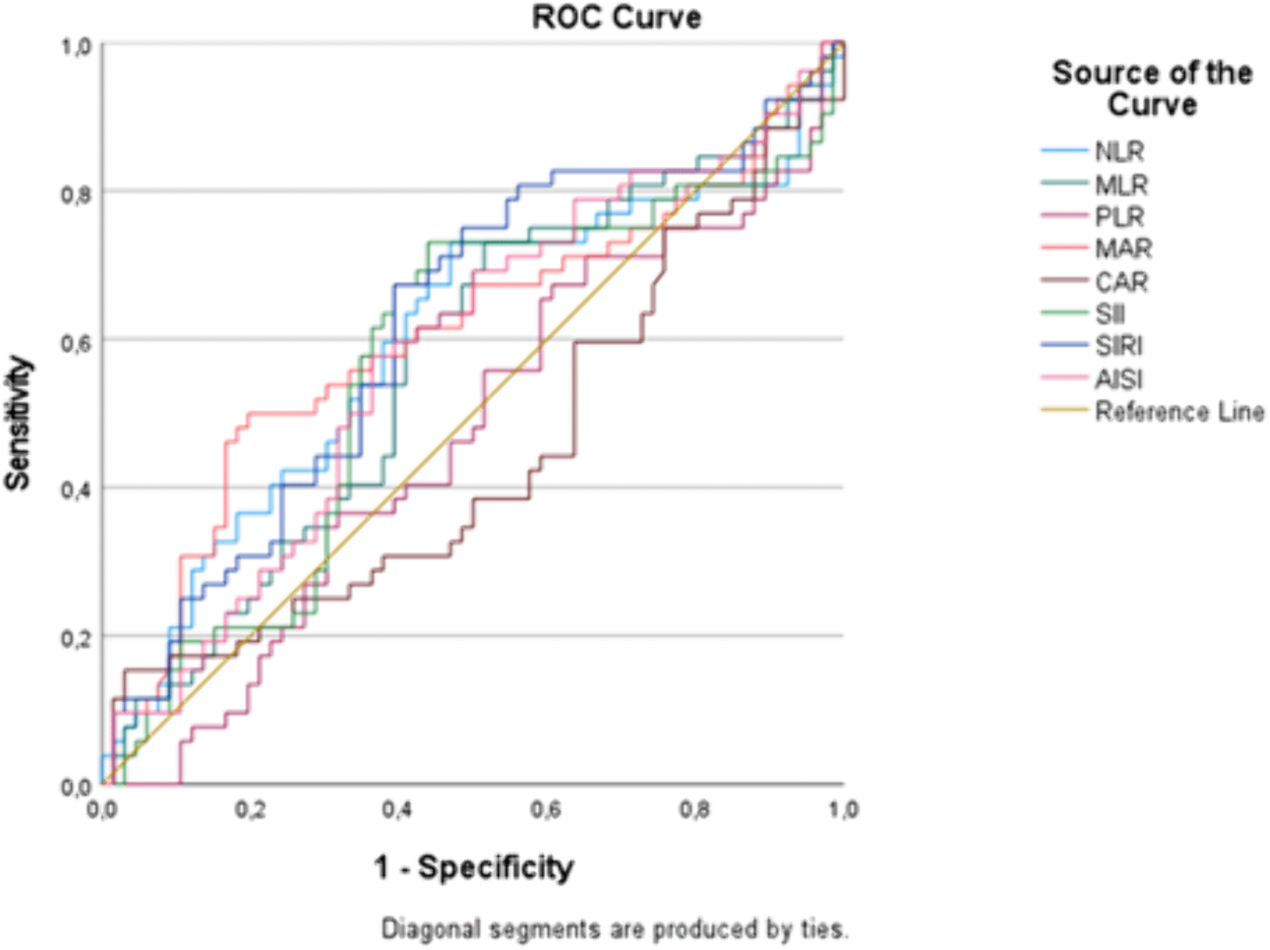
Area under the curve values for 30-day case-fatality.

The ROC curve analysis indicated that none of the inflammatory indices reliably predicted 48-hour or 90-day case-fatality. Conversely, both MAR (AUC = 0.606, p = 0.049) and SIRI (AUC = 0.617, p = 0.029) demonstrated statistically significant but weak predictive performance for 30-day case-fatality (Table 4, Fig 2).

**Table 4:** Predictive performance of indices for 30-day case-fatality classification.

Test Result Variable(s)	Area	Std. Error^a^	p-value	Asymptotic 95% CI	
				Lower Bound	Upper Bound
NLR	0.596	0.055	0.073	0.490	0.703
MLR	0.569	0.054	0.197	0.464	0.674
PLR	0.469	0.054	0.558	0.363	0.574
**MAR**	0.606	0.054	**0.049**	0.499	0.713
CAR	0.443	0.055	0.292	0.336	0.550
SII	0.566	0.055	0.221	0.458	0.674
**SIRI**	0.617	0.053	**0.029**	0.514	0.721
AISI	0.580	0.053	0.137	0.475	0.685

NLR: Neutrofil/Lenfosit ratio, MLR: Monosit/Lenfosit ratio, PLR: Platelet/Lenfosit ratio, MAR: Monosit/Albumin ratio, CAR: C-reactive protein/Albumin Ratio, SII: Systemic immune-inflammatory index, SIRI: Systemic inflammatory response index, AISI: Aggregate index of systemic inflammation. ^**a**^ Under the nonparametric assumption. Significant p-values (<0.05) are shown in bold.

Optimal cut-off values were calculated for the SIRI and MAR indices, which showed significant AUC values. The optimal cut-off for SIRI was 2.49, and for MAR was 0.018. Values exceeding these cut-offs may be interpreted as predictive of case-fatality for both indices, serving as adjunctive tools in risk stratification (Table 5).

**Table 5:** Optimum cutoff values for SIRI and MAR index.

	Cutpoint	Sensitivity (%)	Specificity (%)	PPV (%)	NPV (%)	Youden’s index	AUC	Metric Score
SIRI	2.49	67.31%	60.61%	57.38%	70.18%	0.279	0.617	1.28
MAR	0.018	48.08%	81.82%	67.57%	66.67%	0.299	0.606	1.30

SIRI: systemic inflammatory response index, MAR: monocyte‑to‑albumin ratio, PPV: positive predictive value, NPV: negative predictive value, AUC: area under the receiver operating characteristic curve. *The optimal cutoff of 0.018 was determined using Youden’s index, which prioritizes specificity over sensitivity.

Logistic regression analyses were performed to identify predictors of 30-day and 90-day case-fatality, with both models incorporating age, sex, hemorrhage type (reference: SAH), treatment type, and MAR/SIRI groups as independent variables. The 30-day case-fatality model demonstrated significant predictive value (p = 0.001) with 71.8% accuracy (sensitivity 62.7%, specificity 78.8%). MAR > 0.018 emerged as an independent predictor associated with a 3.57-fold increased case-fatality risk (OR=3.57; 95% CI: 1.44–8.85; p = 0.006) (Table 6).

**Table 6:** Multivariable logistic regression analysis for the prediction of short-term (30-day) case-fatality.

	30-day case-fatality				
	B	p	O.R.	95% CI for EXP(B)	
				Lower	Upper
**Age**	0.032	0.239	1.033	0.979	1.090
**Sex**	0.635	0.149	1.886	0.797	4.462
**Hemorrhage Type (ref: SAH)**		0.254			
**Hemorrhage Type (IPH)**	−0.048	0.953	0.953	0.190	4.791
**Hemorrhage Type (SAH + IPH)**	1.873	0.177	6.505	0.429	98.655
**Treatment type**	0.997	0.229	2.711	0.533	13.772
**MAR (ref: < 0.018)**	1.274	**0.006**	3.573	1.443	8.851
**SIRI (ref: < 2.49)**	0.506	0.264	1.659	0.682	4.035
**Constant**	−1.562	0.447	0.210		

SAH: Subarachnoid hemorrhage, IPH: Intraparenchymal hemorrhage, MAR: Monosit/Albumin ratio. SIRI: Systemic inflammatory response index. MAR: Monocyte/Albumin Ratio, **B: regression coefficient, S.E.: standard error,** OR: odds ratio, CI: confidence interval. Significant p-values (<0.05) are shown in bold.

Similarly, the 90-day case-fatality model showed significant predictive power (Omnibus test p = 0.006) with 66.7% accuracy (sensitivity 58.2%, specificity 74.0%). MAR > 0.018 remained a significant predictor of 90-day case-fatality, corresponding to a 3.18-fold risk increase (OR=3.18; 95% CI: 1.28–7.92; p = 0.013). Age independently predicted 90-day case-fatality (OR=1.06; p = 0.029), while other covariates, including sex, hemorrhage subtypes, treatment type, and SIRI groups, showed no significant associations (Table 7).

**Table 7:** Multivariable logistic regression analysis for the prediction of longer-term (90-day) case-fatality.

	90-day case-fatality					
	B	S.E.	p	O.R.	95% CI for EXP(B)	
**Lower**	**Upper**
**Age**	0.059	0.027	0.029	1.061	1.006	1.119
**Sex**	0.239	0.422	0.571	1.270	0.556	2.902
**Hemorrhage Type (ref: SAH)**			0.244			
**Hemorrhage Type (IPH)**	−0.389	0.774	0.616	0.678	0.149	3.090
**Hemorrhage Type (SAH + IPH)**	1.476	1.353	0.275	4.374	0.308	62.028
**Treatment type**	1.189	0.827	0.151	3.283	0.649	16.599
**MAR (ref: < 0.018)**	1.158	0.466	**0.013**	3.182	1.278	7.924
**SIRI (ref: < 2.49)**	0.145	0.450	0.747	1.156	0.479	2.789
**Constant**	−3.240	2.006	0.106	0.039		

SAH: Subarachnoid hemorrhage, IPH: Intraparenchymal hemorrhage, MAR: Monosit/Albumin ratio. SIRI: Systemic inflammatory response index. MAR: Monocyte/Albumin Ratio, **B: regression coefficient, S.E.: standard error,** OR: odds ratio, CI: confidence interval. Significant p-values (<0.05) are shown in bold.

These findings demonstrate that elevated MAR (>0.018) serves as a consistent prognostic marker for both 30-day and 90-day case-fatality, suggesting its clinical utility extends beyond the acute phase of care. The persistent predictive power of MAR across both time points underscores its potential value for risk stratification in this patient population (Tables 6 and 7).

**Fig 3:**
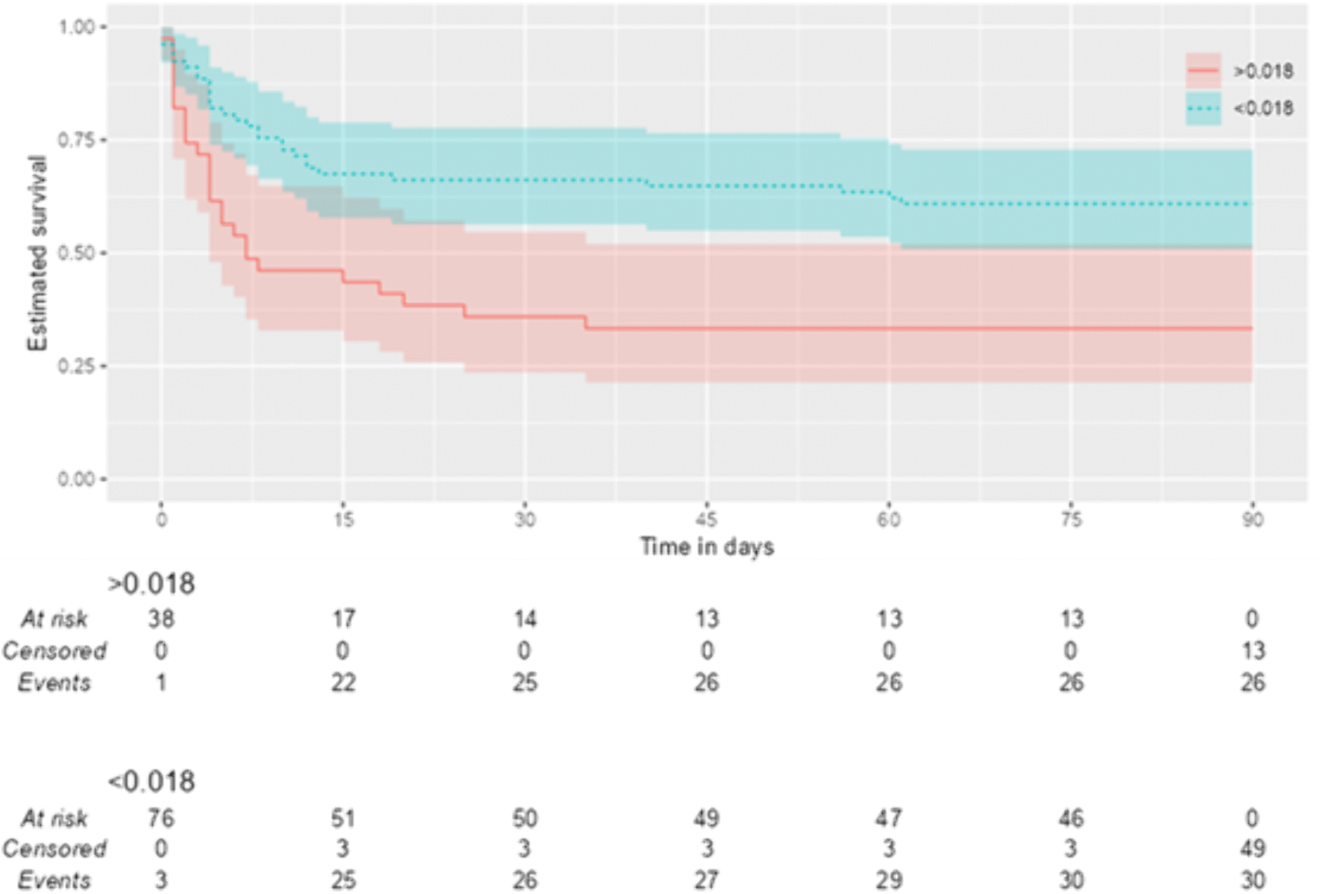
Kaplan-Meier survival curves comparing 90-day survival probabilities by MAR groups.

Cox proportional hazards regression analysis supported these results, demonstrating that patients with MAR ≤ 0.018 had a significantly reduced case-fatality risk over 90 days (HR = 0.44; 95% CI: 0.26–0.74; p = 0.002) (Table 6). When comparing survival rates according to MAR groups at 2, 30, and 90 days, the 2-day survival rate was found to be 74.4% for patients with MAR > 0.018 versus 91.1% for those with MAR ≤ 0.018; the 30-day survival rate was 35.9% for patients with MAR > 0.018 versus 66.2% for those with MAR ≤ 0.018; and the 90-day survival rate was 33.3% for patients with MAR > 0.018 versus 60.9% for those with MAR ≤ 0.018 (Table 8, Fig 3).

**Table 8:** Survival Analysis and Cox Regression Results by MAR Groups.

MAR Group	n (%)	HR (95% CI), p-value	Day 2 Survival (%)	Day 30 Survival (%)	Day 90 Survival (%)
>0.018	39 (33.1)	Reference	74.4% (61.8%−89.4%)	35.9% (23.6%−54.6%)	33.3% (21.4%−52.0%)
**≤0.018**	**79 (66.9)**	**0.44 (0.26–0.74), p = 0.002**	91.1% (85.1%−97.6%)	66.2% (56.4%−77.7%)	60.9% (50.9%−72.9%)

MAR: Monosit/Albumin ratio, CI: confidence interval.

To investigate whether admission inflammatory indices could predict initial hematoma volume, a multiple linear regression analysis was performed. The overall model did not reach statistical significance (F(8,109)=0.833, p = 0.576, R2 = 0.058, adjusted R2=−0.012). However, among the independent variables, MAR was identified as the only significant independent predictor of hematoma volume (B = 1415.79, SE = 672.10, t = 2.107, p = 0.037). Specifically, a one-unit increase in MAR, while holding all other variables constant, was associated with an approximately 1415.79-unit increase in hemorrhage volume, indicating a positive relationship between MAR and the dependent variable. Other indices, including NLR, MLR, PLR, CAR, SII, SIRI, and AISI, did not show a statistically significant contribution to the prediction of hematoma volume (Table 9).

**Table 9:** Multiple linear regression analysis for the prediction of hematoma volume.

Predictor	Estimate (B)	S.E.	95% Confidence Interval		β	df	t	p
**Lower**	**Upper**	
(Intercept)	33.78983	3.9475	25.9659	41.6137	0.0000	109	8.560	**<.001**
NLR	4.86805	3.9033	−2.8682	12.6043	0.6146	109	1.247	0.215
MLR	6.91706	10.5399	−13.9728	27.8069	0.1788	109	0.656	0.513
PLR	−0.06080	0.1022	−0.2633	0.1417	−0.1430	109	−0.595	0.553
MAR	1415.78788	672.0990	83.7094	2747.8663	0.3038	109	2.107	**0.037**
CAR	−4.23733	6.9213	−17.9551	9.4804	−0.0652	109	−0.612	0.542
SII	−0.00942	0.0189	−0.0468	0.0280	−0.2870	109	−0.500	0.618
SIRI	−4.29382	4.0596	−12.3398	3.7522	−0.6610	109	−1.058	0.293
AISI	0.00661	0.0183	−0.0297	0.0429	0.2171	109	0.361	0.719

NLR: Neutrophil-to-Lymphocyte Ratio, MLR: Monocyte-to-Lymphocyte Ratio, PLR: Platelet-to-Lymphocyte Ratio, MAR: Monocyte-to-Albumin Ratio, CAR: CRP-to-Albumin Ratio, SII: Systemic Immune-Inflammation Index, SIRI: Systemic Inflammatory Response Index, AISI: Aggregate Index of Systemic Inflammation, S.E.: Standard Error, β: Standardized regression coefficient, df: degrees of freedom, t: t-statistic, Significant p-values (<0.05) shown in bold.

**Fig 4:**
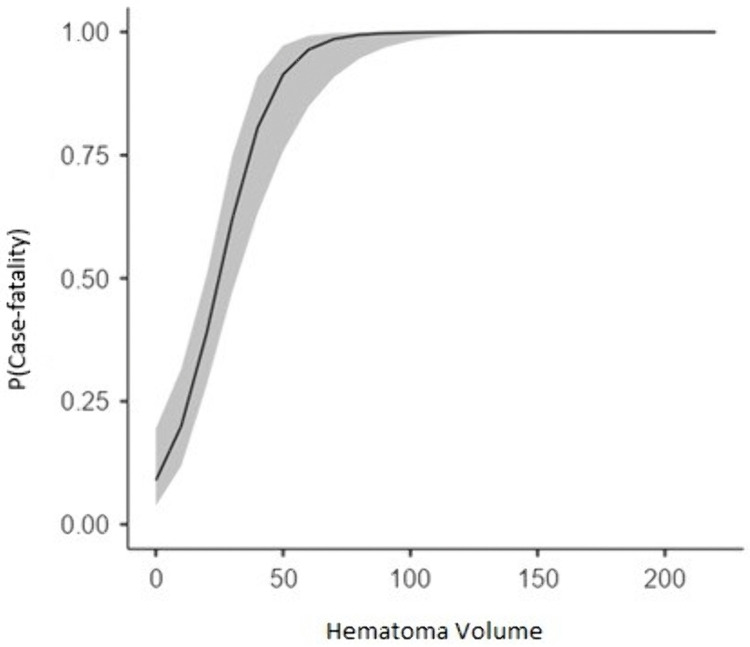
Predicted probability of case-fatality relative to hemorrhage volume.

Logistic regression analysis was conducted to evaluate the influence of hemorrhage volume on case-fatality. The model was statistically significant (χ²(1) = 60.2, p < 0.001) and demonstrated strong predictive fit (McFadden R² = 0.369). The results showed that each one-unit increase in hemorrhage volume significantly elevated the odds of case-fatality by 9.8% (OR = 1.098, 95% CI: 1.058–1.140, p < 0.001; Table 10). The positive regression coefficient (B = 0.0937, S.E. = 0.0193, Z = 4.86) indicates that as hemorrhage volume increases, the probability of case-fatality rises significantly. This relationship is further illustrated in Fig 4, which shows the sharply increasing probability of case-fatality as hemorrhage volume expands.

**Table 10:** Logistic regression model predicting fatality based on hemorrhage volume.

Predictor	Estimate	S.E.	Z	p	Odds ratio	95% Confidence Interval	
						Lower	Upper
Intercept	−2.3225	0.4612	−5.04	0 < .001	0.0980	0.0397	0.242
Hematoma volume (mL)	0.0937	0.0193	4.86	**0 < .001**	1.0982	1.0575	1.140

CI: confidence interval, OR: odds ratio, S.E.: standard error.

Collectively, these findings illustrate the possible role of systemic inflammatory indices, particularly MAR, in predicting short- and medium-term case-fatality among elderly patients with spontaneous ICH.

## Discussion

The primary contribution of this study is the identification of admission MAR and SIRI as novel, readily available prognostic biomarkers for predicting case-fatality in geriatric patients with ICH. This focus is essential because, within a cohort already restricted to the elderly, chronological age per se often acts as a weak prognostic discriminator. As demonstrated by Batista et al. (2021), the risk of short-term death in very old patients is primarily driven by factors such as hematoma volume, level of consciousness, and functional reserve, rather than age itself [17]. Following the initial hemorrhage, disruption of the BBB triggers a cascade of inflammatory and oxidative stress processes, driven by activation of circulating neutrophils and monocytes, contributing to perihematomal edema, oxidative stress, and neuronal apoptosis [6,18]. Given the dual role of the immune response in both tissue repair and damage potentiation, inflammatory indices hold significant prognostic weight. Our results indicate that while both MAR and SIRI were linked to 30-day case-fatality, MAR remained an independent predictor for both 30- and 90-day outcomes after adjusting for clinical covariates. The most striking finding is that MAR provided predictive utility for both short- and medium-term case-fatality, whereas SIRI showed only a univariate association with 30-day outcomes. Although the individual discrimination of these markers was modest, it is important to recognize that established ICH scores often demonstrate ceiling effects when applied to exclusively older populations, limiting their predictive sensitivity in this age group [19]. Consequently, rather than being used as stand-alone tools, these readily available biomarkers should be integrated into existing prognostic models to refine early risk stratification and improve overall predictive accuracy. Our study represents one of the few investigations to evaluate these indices specifically in a geriatric ICH cohort, and integrating MAR into established scores, such as the ICH Score, could further enhance predictive power [20]. This approach provides a more comprehensive perspective, helping to avoid defeatist clinical attitudes and ensuring that optimal acute treatment is offered to elderly patients. Nevertheless, the low sensitivity of MAR precludes its use as a standalone prognostic tool; rather, it should serve as an adjunctive marker to refine risk stratification in combination with established clinical predictors.

The prognostic importance of NLR and other compared biomarkers in ICH is well-established in the literature, with elevated NLR shown to correlate with hematoma expansion and poor functional outcomes [21,22]. Accumulating evidence indicates that monocytes are particularly critical for inflammatory injury following ICH [8]. Albumin is regarded as a negative acute-phase protein, which declines during inflammation; conversely, albumin exerts neuroprotective effects by maintaining vascular integrity and reducing oxidative stress [8]. Low serum albumin levels have been associated with poor functional outcomes in ICH patients [8,23]. Furthermore, albumin levels reflect nutritional status and systemic resilience, which are determinants of survival in elderly patients [23,24]. MAR integrates these two biologically relevant parameters into a single index. In our cohort, higher admission MAR was associated with larger initial hematoma volume, suggesting that the systemic inflammatory‑nutritional state captured by MAR may relate to the mechanical severity of the primary hemorrhage. While hematoma volume remains a primary determinant of case-fatality, with its impact often influenced by anatomic location [25], the independent predictive value of MAR suggests it captures pathological processes beyond simple mass effect. Our findings extend the observations of Fu et al. (2024), who identified MAR as an independent predictor of hematoma expansion, which is also associated with poor prognosis [8], by demonstrating that this systemic vulnerability is already manifest at admission, rather than solely appearing during later hematoma expansion.

The prognostic significance of MAR in our geriatric ICH cohort is further reinforced by recent evidence in the context of acute ischemic stroke (AIS). Lu et al. (2025) demonstrated that a higher admission MAR is an independent predictor of poor 3-month functional outcomes in AIS patients, particularly those with large-artery atherosclerosis [26]. Notably, their methodology follows a similar exclusion pattern to our own; they also excluded patients with active infections, hematologic disorders, and hepatic insufficiency to ensure the MAR specifically reflected the stroke-related inflammatory state rather than pre-existing systemic confounders. This suggests that the inflammatory-nutritional profile captured by MAR represents a common pathological thread across different stroke types. The importance of these factors is underscored by the fact that a pro-inflammatory environment, which is frequently driven by metabolic comorbidities, can amplify the secondary inflammatory cascade following the initial brain injury. In this state, elevated monocyte levels reflect systemic immune activation and their subsequent infiltration into the brain parenchyma, where they interact with resident microglia to drive neuroinflammation. Simultaneously, low albumin levels indicate a depletion of the body’s antioxidant and neuroprotective reserves, leaving the brain more vulnerable to this inflammatory onslaught. Consequently, MAR may serve as an indicator of a patient’s systemic resilience and the intensity of the post-stroke inflammatory response in both hemorrhagic and ischemic events [8,26].

In our cohort, NLR did not emerge as an independent predictor of case-fatality. This may be attributed to the advanced age of the study population and differences in baseline comorbidities, such as high prevalence of hypertension and anticoagulant therapy, which can modulate systemic inflammatory responses [27]. Our findings align more closely with recent results by Li et al., indicating SIRI is a stronger predictor than NLR [11]. Elevated SIRI levels have been associated with increased perihematomal edema and delayed recovery in ICH [11]. While higher SIRI values demonstrated moderate predictive power for 30-day case-fatality, consistent with prior evidence suggesting it reflects secondary neuroinflammation via neutrophil-mediated tissue damage and lymphocyte depletion [11]. However, its lack of significance in multivariate analyses implies MAR may be a superior biomarker in elderly patients.

This perspective is reinforced by the large-scale analysis of Zhao et al. (2024), which compared multiple inflammatory indices in critically ill ICH patients with a mean age comparable to our cohort [28]. In their study, NLR demonstrated the highest predictive value for intensive care unit (ICU) case-fatality, followed by SIRI, with multivariable analysis confirming NLR as a significant independent predictor alongside albumin. While NLR did not retain independent significance in our multivariable model due to a discrepancy potentially attributable to differences in outcome measures (ICU vs. 30/90-day case-fatality), patient selection, and sample size, both studies converge on several key points. First, both identify low albumin as an independent risk factor for case-fatality, underscoring the prognostic importance of nutritional status and systemic resilience. Second, both demonstrate that composite inflammatory indices reflecting multiple immune cell lineages outperform individual parameters. Notably, our study extends these findings by identifying MAR as a particularly robust predictor in the geriatric population. The superior performance of MAR in our elderly cohort may reflect the heightened vulnerability of older patients to the dual insults of inflammation and malnutrition, a synergy not captured by NLR or SIRI alone [28].

### Strengths and limitations

This study has several strengths and limitations that warrant consideration. Strengths include that this is one of the few investigations to evaluate systemic inflammatory indices specifically in a geriatric spontaneous ICH cohort. The inclusion of detailed volumetric analysis using validated methods (ABC/2 for intraparenchymal hemorrhage, compartmental ABC/2 for subarachnoid hemorrhage) allowed adjustment for hematoma volume.

This study has several limitations that warrant consideration. First, a more thorough adjustment was not possible due to the lack of key ICH prognosticators, such as admission GCS and intraventricular extension. Second, the number of events (case-fatalities) at 48 hours was low, limiting statistical power; therefore, non‑significant findings at 48 hours should be interpreted with caution as they may reflect type II error. Higher event rates at 30 days and 90 days provided greater sensitivity for those time points. Third, our multivariable logistic models obtained an events-per-variable ratio of roughly 7–8, which is below the standard rule-of-thumb of 10 and may inflate effect estimates, given there were 52 and 56 case-fatality events at 30 and 90 days, respectively. Fourth, the derived cut-offs for MAR and SIRI require external validation because they were determined in-sample using Youden’s index. Additionally, the MAR cutoff showed low sensitivity, but high specificity, meaning that MAR may be useful as a “rule in” marker to identify a high-risk subgroup, but it cannot be used alone for prognostic decisions. Fifth, the exclusion of 38 patients (approximately 11% of the initially screened population) due to relocation or incomplete follow-up data introduces potential attrition bias, as these individuals may have represented a different clinical or functional subgroup than those retained in the cohort. Such systematic differences could have influenced the observed associations between inflammatory indices and case-fatality. Finally, generalizability is limited by the retrospective, single-center design, and prospective, multicenter studies with complete follow-up are needed to confirm these findings.

As a conclusion, SIRI performs worse and is only associated with 30-day case-fatality in older patients with spontaneous ICH, while admission MAR is independently associated with 30- and 90-day case-fatality. Prior to routine clinical adoption, MAR should be considered an adjunctive marker that requires external validation due to its data-driven thresholds and modest discrimination.

## Supporting information

S1 File Raw data in comma-separated values (CSV) format and the corresponding codebook (PDF), compressed in a single RAR archive. (RAR)
